# RF catheter ablation of AVNRT in a patient with interrupted inferior vena cava anomaly with hemiazygos continuity with persistent left superior vena cava

**DOI:** 10.1186/s13019-024-02899-1

**Published:** 2024-07-15

**Authors:** Ali Bozorgi, Faezeh Tabesh, Mansour Jahangiri, Parham Rabiei, Entezar Mehrabi Nasab

**Affiliations:** 1 Department of Cardiology, School of Medicine, Tehran Heart Center, Tehran University of Medical Sciences, Tehran, Iran; 2 Cardiac Rehabilitation Research Center, Cardiovascular Research Institute, Isfahan University of Medical Sciences, Isfahan, Iran; 3 Department of Interventional Cardiology, Milad Hospital, Tehran, Iran; 4 Rajaie Cardiovascular Medical and Research Center, Iran University of Medical Sciences, Tehran, IR Iran; 5 Department of Cardiology, School of Medicine, Valiasr Hospital, Zanjan University of Medical Sciences, Zanjan, Iran; 6 Tehran Heart Center, North Kargar Street, Tehran, Iran

**Keywords:** RF ablation, AVNRT, IVC interrupted, Persistent LSVC

## Abstract

Intrahepatic interruption of the inferior vena cava (IVC) with continued hemizygous is a very rare abnormality and sometimes it may be accompanied by other cardiovascular abnormalities. Continuation of the hemizygous vein draining into the right atrium through the left superior vena cava (LSVC) is much rarer. In this paper, we have presented a patient who had simultaneous IVC interrupted with persistent LSVC and suffered from Atrioventricular nodal reentrant tachycardia (AVNRT). Finally, radiofrequencies (RF) catheter ablation for AVNRT was successfully performed through a left subclavian vein access.

## Introduction

Intrahepatic IVC interruption with azygos/hemiazygos continuation is a very rare anomaly [1, 2] and hemiazygos vein continuation draining into the right atrium via a persistent LSVC is even rarer [3]. This abnormality is often manifested as an isolated abnormality, but sometimes it may be association by other congenital abnormalities such as left isomerism, heart or spleen abnormalities. The prevalence of azygos continuation of the IVC is 0.3% if it is isolated, and it is about 0.6-2% if it is association by other anomalies [4]. In this article, we report a patient with IVC interrupted anomaly and hemiazygos continuity with persistent LSVC and suffering from AVNRT at the same time.

## Case presentation

**Fig. 1:**
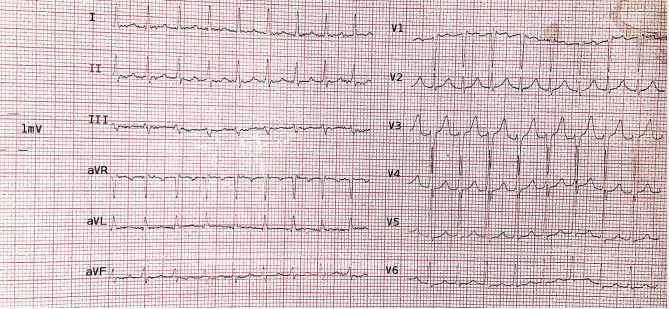
Narrow QRS complex tachycardia in favor of AVNRT

**Fig. 2:**
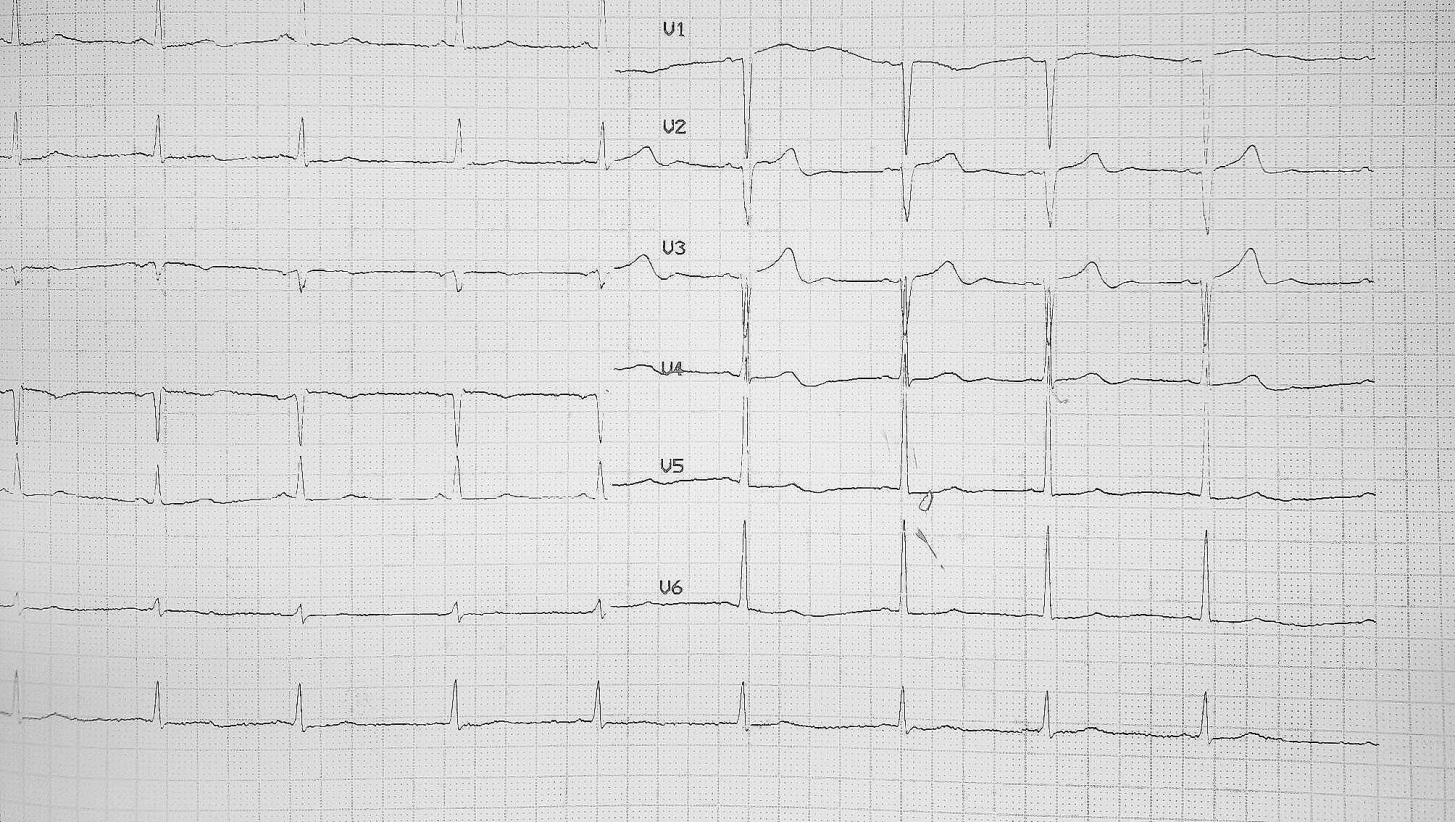
ECG of normal sinus rhythm

A 44-year-old woman came to the emergency room of our hospital with a complaint of palpitations. The patient has repeatedly experienced throbbing attacks similar to this attack in the last two years and despite receiving beta blocker, the arrhythmia attacks were repeated. ECG of 12 leads was immediately prepared (Fig. 1), regular narrow QRS complex tachycardia with normal axis was observed, which was in favor of paroxysmal supraventricular tachycardia (PSVT). 12 mg of adenosine was injected and the arrhythmia stopped immediately. After controlling the patient’s rhythm (Fig. 2) and stabilizing it, according to the history of dyspnea, a chest X-ray was prepared, in which cardiomegaly was seen. There was no evidence in favor of pulmonary edema, pleural effusion or pericardial effusion. Then transthoracic echocardiography (TTE) was performed and showed sever dilated left ventricular (LV) and sever systolic disfunction (LVEF = 30%), global hypokinesia. Mild right ventricular (RV) enlargement and mild systolic dysfunction. moderate functional mitral regurgitation (MR) and mild tricuspid regurgitation (TR). Mild pulmonary artery hypertension (PAP = 30 mmHg). The coronary sinus was dilated (18 × 20 mm) with the possibility of persistent LSVC, agitated saline injection into the left antecubital IV showed the entrance of contrast in to the dilated CS before right sided chambers.

**Fig. 3:**
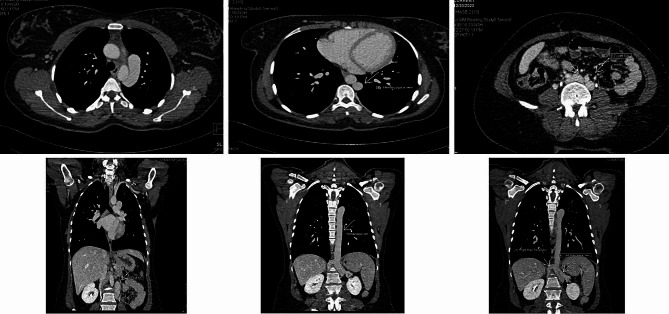
Chest and Abdominal MDCTA in favor of IVC interrupted

Suspected of PSVT, the patient was a candidate for electrophysiology study (EPS) and RF catheter ablation. We decided to perform EPS through the right transfemoral vein access. But unfortunately, the catheters were not able to pass through the IVC. Venography was performed and the evidence was in favor of IVC interrupted. The procedure was terminated and for definitive diagnosis of venous anomaly, the patient became a candidate for Multidetector Computed Tomography (MDCT) of chest, abdomen and heart. Coronary MDCT scan showed normal epicardial coronary artery. Abdominal MDCT scan showed that IVC was interrupted in hepatic segment with continued hemiazygos. Then the hemiazygos drains into the persistent LSVC. Right SVC was absent incredibility (Fig. 3).

In electrophysiology (EP) lab, we were able to position two 6 F (quadripolar high in right atrium and decapolar in coronary sinus) catheters within the left femoral artery access.

**Fig. 4:**
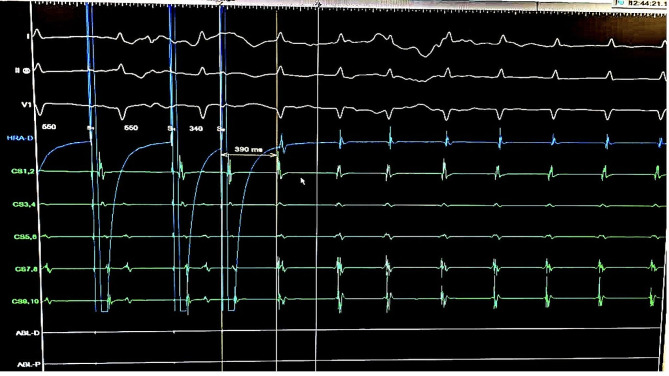
Atrial stimulation and easily inducible AVNRT

**Fig. 5:**
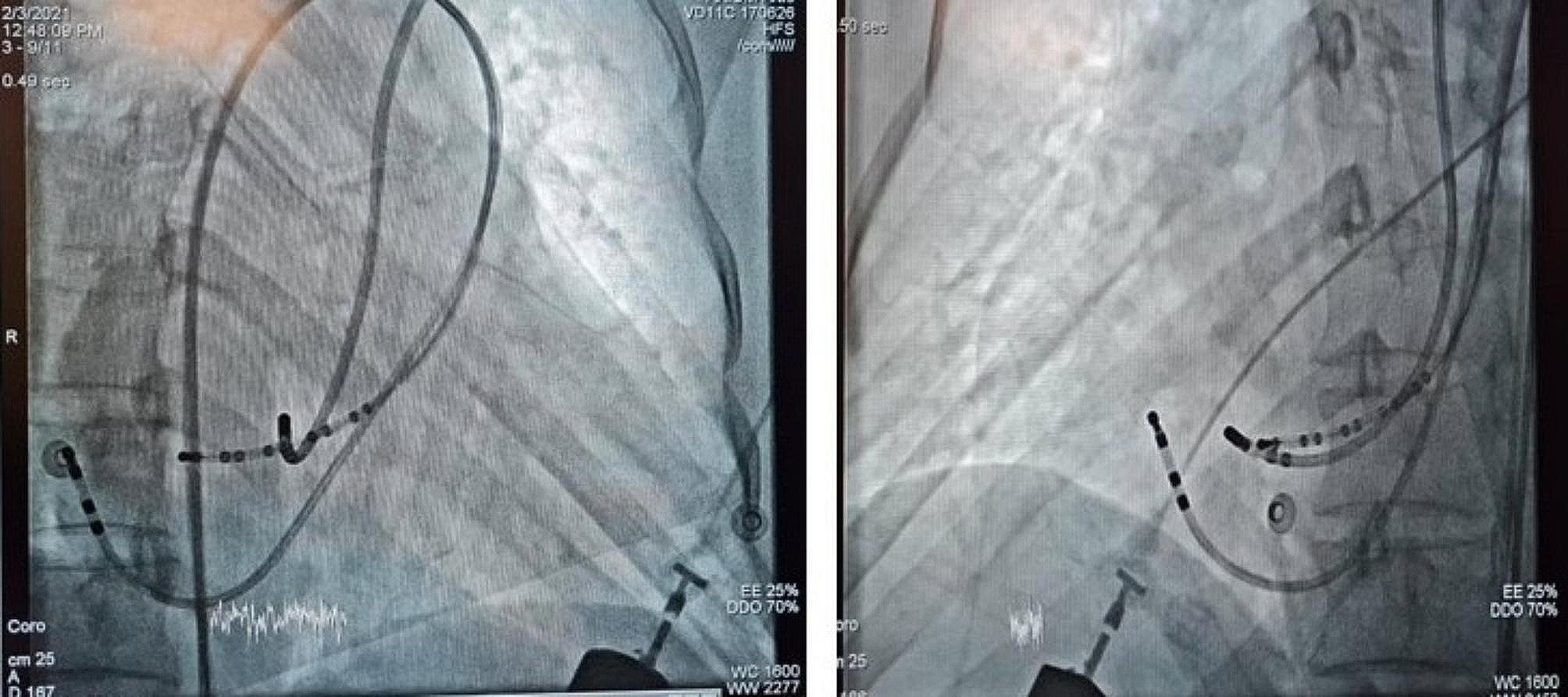
Position of catheters during mapping and RF ablation

**Fig. 6:**
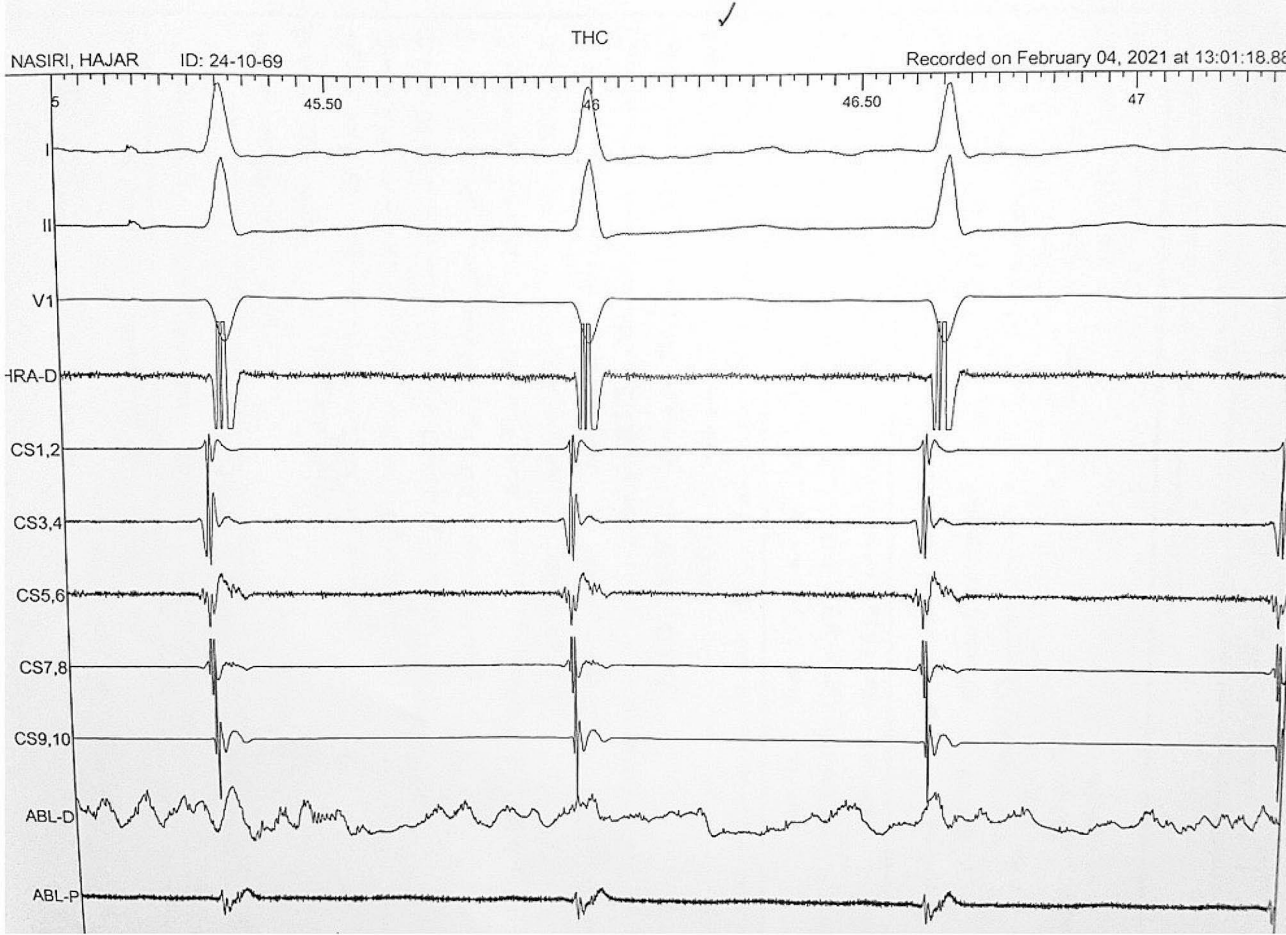
Junctional rhythm during successful application of RF at the slow pathway site **Video 1.** A ablation catheter was positioned in the right side His bundle to recognize His area and then repositioned in slow pathway area through a left subclavian vein access

Catheters were placed after the femoral vein through the hemiazygos vein and then LSVC in the coronary sinus and right atrium. We were initially unable to place the RV catheter. Then by programed Atrial extra stimulation a regular narrow QRS complex tachycardia with 1: 1 VA association and short VA interval (10 ms) and concentric retrograde atrial activation was induced, in favor of AVNRT,. Given that the AVNRT has a very short VA interval and long AV interval that was compatible with typical AVNRT (Fig. 4). A 4 mm non-irrigated bidirectional curve ablation catheter was positioned in the right side His bundle to recognize His area and then repositioned in slow pathway area through a left subclavian vein access (Fig. 5, video 1). The advantage of this approach was the better manipulation and reposition of the catheter due to the shorter and less tortuous course. RF current was delivered to slow pathway target area. Abundant Junctional rhythm developed during all energy applications (Fig. 6). Atrial burst pacing and up to three atrial extra stimuli did not induce any arrhythmia after ablation even with isoproterenol infusion. The patient was observed for 12 h and then discharged with a good general condition and was recommended to see a heart failure specialist.

## Discussion

Congenital malformations of the IVC are extremely rare. The most common malformation of the IVC is the subhepatic interruption of the IVC. In the embryonic period, the IVC includes the hepatic, perirenal, renal, and postrenal segments. Non-connection of hepatic and pararenal parts leads to interruption of subhepatic IVC. Sometimes subhepatic IVC continues as azygos vein or hemizygous vein to LSVC [3, 5, 6]. Failure to regression of left anterior cardiac vein or vein of marshal leads to creation of LSVC [7]. IVC interruption with azygos/hemiazygos continuation is a very rare anomaly [1, 2]. This anomaly is often described as a congenital heart disease with heterotaxy (cardiosplenic) syndrome [6, 8]. But sometimes it may occur in isolation without other congenital abnormalities. When it is an isolated event without heart disease, it is likely to be overlooked due to the lack of symptoms in early life and is often detected incidentally during radiological examination or vascular interventions. Abnormalities in systemic venous return may be associated with cardiac conduction system abnormalities and therefore lead to bradyarrhythmias or tachyarrhythmias. The occurrence of arrhythmias may be due to primary or secondary causes. Dilation of the coronary sinus can cause tension on the atrioventricular node or the bundle of His and therefore cause arrhythmia [9]. Morgan et al. reported abnormal venous drainage in 4% of 300 patients who underwent an EPS before implantable pacemaker defibrillator or implantable cardioverter implantation [10]. Jongmin et al. reviewed all patients with supraventricular arrhythmia who underwent EPS or RF ablation during a 10-year study period, and about 0.27% of them had PLSVC and interestingly, the most common supraventricular arrhythmia (SVA) among PLSVC patients was AVNRT. Among patients with AVNRT, an SP in the CS was significantly more frequent in patients with PLSVC than in those without PLSVC (13). The success rate of catheter ablation was 82% in SVA patients with PLSVC. There were no procedure-related complications [11]. In the case we have reported, the arrhythmia that was present was AVNRT and was successfully ablated in slow pathway, confirming the results of Jongmin study. But the important point is that due to the abnormalities in the cardiovascular structure of these patients, they are prone to recurrence of arrhythmias. Therefore, it is strongly recommended that in patients with abnormal venous return, a general evaluation, including complete imaging and electrophysiological studies, must be performed. It is recommended that annual follow-up be performed and any deviation from baseline be a criterion for cardiac evaluation [10]. However, performing catheter ablation in patients with cardiac or vascular anomalies is difficult, sharing your experiences with others can help doctors to better manage their patients in the future.

## Data Availability

No datasets were generated or analysed during the current study.
